# Supplementary material for: SARS-CoV-2 RNA Extraction Using Magnetic Beads for Rapid Large-Scale Testing by RT-qPCR and RT-LAMP
Source: Viruses. 2020 Aug 7;12(8):863. doi: 10.3390/v12080863 (PMC7472728; doi:10.3390/v12080863)
Supplement: Supplementary file 1 [file viruses-12-00863-s001.pdf]

Supplementary Materials

SARS-CoV-2 RNA Extraction Using Magnetic Beads for Rapid Large-Scale Testing by RT-qPCR and RT-LAMP

Steffen Klein [1,2,†], Thorsten G. Müller [1,†], Dina Khalid [1], Vera Sonntag-Buck [1], Anke-Mareil Heuser [1], Bärbel Glass [1], Matthias Meurer [3,4], Ivonne Morales [5], Angelika Schillak [1], Andrew Freistaedter [1], Ina Ambiel [1], Sophie L. Winter [1,2], Liv Zimmermann [1], Tamara Naumoska [1], Felix Bubeck [1], Daniel Kirrmaier [3,4], Stephanie Ullrich [1], Isabel Barreto Miranda [1], Simon Anders [3], Dirk Grimm [1,6], Paul Schnitzler [1], Michael Knop [3,4,7], Hans-Georg Kräusslich [1,6], Viet Loan Dao Thi [1,2], Kathleen Börner [1,6,*], and Petr Chlanda [1,2,*]

† Equal contribution.

* Correspondence: petr.chlanda@bioquant.uni-heidelberg.de; kathleen.boerner@bioquant.uni-heidelberg.de

1. List of Materials and instruments for magnetic beads extraction

Reagent	Company	Material #
Guanidinium thiocyanate (GTC)	Carl Roth	0017
Sodium citrate	Carl Roth	3580
Triton X-100	Carl Roth	3051
Glycogen (5 µg/µl)	Invitrogen	AM9510
SiMAG-N-DNA magnetic beads (100 µg/µl)	Chemicell	1104-5
Nuclease-free water	Life Technologies	AM9938
Ethanol abs.	Sigma-Aldrich	32205
Diethyl pyrocarbonate (DEPC)	Sigma-Aldrich	D5758
Dithiothreitol (DTT)	Carl Roth	6908

Consumable	Company	Material #
Masterblock, 96 well, PP, 0.5 ml, V-bottom	Greiner	786261
twin.tec 96-well PCR Plate, skirted, colorless	Eppendorf	951020401
Molded Polypropylene Reservoir for Liquidator	V&P Scientific	VP 576D
Pipette Tips LQR LTS 200 µl F 960/10	Mettler Toledo	17010646
Grip-Tips 300 µl long, sterile with filter	Integra Bioscience	4485

Equipment	Company	Material #
Magnet Plate for 96-well deep well plates	Magtivio	MDMG0013
Liquidator 96 Manual Pipetting System	Mettler Toledo	17010335
Magnetic rack DynaMag™-2 Magnet	Thermo Fisher Scientific	12321D
1.25 ml 8-multichannel dispenser pipette	Eppendorf	
Vortexer	IKA	MS3

2. Detailed step-by-step procedure for RNA extraction using magnetic beads

2.1 Prepare stock solutions

DEPC-treated water (1000 ml)

- Add 1 ml DEPC to 1000 ml MilliQ (Merck) purified water
- Shake vigorously
- Autoclave for 15 min at 121 °C to deactivate DEPC
- Store at room temperature

70% Ethanol (1000 ml)

- Mix 700 ml of EtOH abs. and 300 ml of DEPC-treated water

GTC lysis buffer stock (1000 ml)

- Dilute in 400 ml DEPC-treated water at room temperature:
  - 590.8 g GTC (5M final conc.)
  - 7.35 g Sodium citrate (25 mM final conc.)
  - 10 ml Triton X-100 (1% final conc.)
  - 20 ml DTT (2M stock) (40 mM final conc.)
- Adjust pH to 8 using NaOH
- Fill up to 1000 ml with DEPC-treated water
- Aliquot and store at 4°C *(Note 1)*

Complete GTC lysis buffers for 1x 96-well plate

- Just before RNA extraction, prepare complete GTC lysis buffer
- Check that no crystals are present in the GTC lysis buffer stock
- Mix the following components
  - 14.4 ml GTC lysis buffer stock
  - 60 µl Glycogen (20 µg/ml final conc.)
  - 535 µl RT-qPCR internal control (5 µg/sample final conc.)

SiMAG-N-DNA beads dilution for 1x 96-well plate

- Just before RNA extraction, prepare magnetic bead dilution
- Vortex tube containing magnetic bead stock
- Transfer 1.1 ml magnetic bead stock to a 1.5 ml Eppendorf tube
- Wash magnetic beads with RNase free water (3×)
  - Place on a magnetic rack for 2 min
  - Remove the supernatant
  - Add 1 ml RNase-free water
  - Mix well by vortexing
  - Perform this washing step three times in total
- Prepare magnetic bead dilution in EtOH abs.
  - 19 ml EtOH abs.
  - 1 ml SiMAG-N-DNA (5 µg/µl final conc.)
- Vortex dilution well before use

2.2 Perform RNA extraction using magnetic beads

Preparation and deactivation of pharyngeal swab patient samples

1. For each patient sample, prepare one 1.5 ml tube containing 140 µl freshly prepared complete GTC lysis buffer
2. Transfer 140 µl of the pharyngeal swab patient sample using Grip-Tips (300 µl, long) to the previously prepared 1.5 ml tubes containing complete GTC lysis buffer
3. Vortex for 10 sec
4. Briefly spin and incubate for 10 min at room temperature
5. Transfer lysates (280 µl) of all patient samples into a 0.5 ml 96 deep-well plate

RNA binding to magnetic beads

1. Vortex freshly prepared magnetic beads dilution
2. Add 200 µl of magnetic beads using a multichannel pipette into each well of the 96 deep-well plate containing the lysates. Pipette up and down 10 – 15×
3. Incubate 96 well plate on a Vortexer for 8 min
4. Resuspend sedimented beads using Liquidator (10×)
5. Incubate for an additional 7 min on Vortexer
6. Place the deep-well plate on a magnet plate for 10 min
7. Ring pellets should be formed (Figure 1E)

Ethanol washing (3×)

1. Remove supernatant
2. Remove the deep-well plate from the magnet plate
3. Add 200 µl 70% EtOH to each well using Liquidator
4. Resuspend (10×) sedimented beads using Liquidator
5. Check that a brownish suspension is formed (Figure 1B)
6. Place deep-well on magnet plate
7. Incubate until ring pellets are formed (ring pellets will form quickly at this step, approx. 1 min)
8. Perform this washing step three times

Water washing

1. Remove supernatant using Liquidator
2. Carefully rinse the ring pellets with 60 µl of RNase-free water using Liquidator while keeping the deep-well plate on the magnet plate *(Note 2)*
3. Discard the 60 µl of RNase-free water and immediately proceed to the elution step

RNA Elution

1. Remove the plate from the magnet plate
2. Add 60 µl of RNase-free water to each well using Liquidator
3. Resuspend (10×) sedimented beads using Liquidator
4. Inspect the plate: all pellets should be resuspended, if not use a 200 µl pipet to resuspend the pellet in individual wells
5. Place the deep-well plate on Vortexer for 5 – 10 min
6. Place deep-well plate on magnet plate
7. Incubate until ring pellets are formed (ring pellets will form quickly at this step, approx. 1 min)
8. Transfer 55 µl of the eluate to new 96 well PCR plate using Liquidator
9. Place the 96 well PCR plate on magnet plate

10. Incubate until ring pellets are formed (ring pellets will form quickly at this step, approx. 1 min)
11. To remove any residual beads, transfer 50 µl of the eluate to new 96 well PCR plate using Liquidator

Note 1. Crystals may form in the buffer after a prolonged storage at 4°C.

Note 2. This step assures that all residual ethanol is removed and will not interfere with qPCR, some beads and RNA might be lost.

Supplementary Table S1. Specificity and sensitivity of qPCR as a pool of 3 independent magnetic bead RNA extractions. To determine specificity and sensitivity QIAcube qPCR was used as a reference; CI = confidence intervals.

CT range	40-35	35-30	30-25	25-0
True positive	7	24	26	25
True negative	73	73	73	73
False positive	3	3	3	3
False negative	6	0	0	0
Sensitivity	54%	100%	100%	100%
Specificity	96%	96%	96%	96%
Sensitivity 95% CI	25 – 81%	86 – 100%	87 – 100%	86 – 100%
Specificity 95% CI	89 - 99%	89 - 99%	89 - 99%	89 - 99%

Supplementary Table S2. Sensitivity and specificity of colorimetric RT-LAMP after magnetic bead RNA extractions. To determine sensitivity and specificity QIAcube qPCR was used as a reference; CI = confidence intervals.

CT range	40-35	35-30	30-25	25-0
True positive	0	4	14	15
True negative	29	29	29	29
False positive	0	0	0	0
False negative	6	12	1	0
Sensitivity	0%	25%	93%	100%
Specificity	100%	100%	100%	100%
Sensitivity 95% CI	0 – 46%	7 – 52%	68 – 100%	78 – 100%
Specificity 95% CI	88 – 100%	88 – 100%	88 – 100%	88 – 100%

Supplementary Table S3. Sensitivity and specificity of fluorescent RT-LAMP after magnetic bead RNA extractions. To determine sensitivity and specificity QIAcube qPCR was used as a reference; CI = confidence intervals.

CT range	40-35	35-30	30-25	25-0
True positive	0	11	15	15
True negative	29	29	29	29
False positive	0	0	0	0
False negative	6	5	0	0
Sensitivity	0%	69%	100%	100%
Specificity	100%	100%	100%	100%
Sensitivity 95% CI	0 – 46%	41 – 89%	78 – 100%	78 – 100%
Specificity 95% CI	88 – 100%	88 – 100%	88 – 100%	88 – 100%
